# Application of Phytosociological Information in the Evaluation of the Management of Protected Areas

**DOI:** 10.3390/plants12020406

**Published:** 2023-01-15

**Authors:** Jaime F. Pereña-Ortiz, Ángel Enrique Salvo-Tierra, Daniel Sánchez-Mata

**Affiliations:** 1 Department of Botany and Plant Physiology, Faculty of Sciences, University of Malaga, Campus de Teatinos, Bulevar Louis Pasteur s/n, 29010 Malaga, Spain; 2 Harvard University Herbaria, Department of Organismic and Evolutionary Biology, Harvard University, 22 Divinity Avenue, Cambridge, MA 02138-2094, USA

**Keywords:** plant communities, syntaxonomic distinctness, rarefaction, conservation priority, legal protection, protected area

## Abstract

The classification system of plant communities using phytosociological methods can be applied to their conservation in protected areas, as well as in establishing adequate protections and granting legal status to such areas. A new integrative index is developed to classify plant communities for the evaluation of the conservation status of protected areas, obtained from the product of three statistical indices of diversity: Syntaxonomic Distinctness, Rarefaction and Areas Prioritisation, which has been named DRA (acronym of the three indices used). The DRA is used to assess whether the status granted to Protected Areas matches the values provided by the plant communities within them and which were the basis for the identification and description of the Habitats of Community Interest (Habitats Directive—92/43/CEE). The proposed method was applied to the network of protected natural areas on the Andalusian coast, including 14 areas with different protection status, where, once the plant communities they contain were identified, the DRA index was applied to each of them and compared with the Legal Protection Index, i.e., the current protection regime; it becomes clear, objectively, that not all the statuses assigned, whether the IUCN criteria or those of the Andalusian government, correspond to the real levels of protection they should have on the basis of their plant communities.

## 1. Introduction

The analysis of landscape from the phytosociological point of view is a valuable tool for its comprehensive study, including its dynamism and heritage value. The common methodologies used in phytosociology are considered as an optimal choice in environmental management assessments of habitats, as has been recognized for decades [1,2,3,4,5,6,7,8]. It is based on floristic inventories of homogeneous areas and the evaluation of the taxa present according to their abundance and dominance. This method has proven very useful in obtaining knowledge on vegetation and its dynamics over increasingly large territories [9,10]. Despite the implicit subjectivity of such information, the enormous amount that has accumulated over the past century is currently viewed as an extraordinary database susceptible to statistical and multivariate analysis, using the inventory as a working unit. Researches have shown that these observations can be treated with a high degree of confidence [5,11,12]. The use of taxa and plant communities as indicators in land-use planning and their application in natural environment conservation policies is accepted in several countries, insofar as they are in themselves the object of such protection [13,14]. Some studies have applied the information contained in the study of vegetation (habitats) and their cartographic representation to territorial biological assessment criteria for natural areas [15,16,17]. Loidi (2008) [18] estimated the environmental or naturalistic value of natural spaces, for which he proposed an evaluation methodology based on the application of indices that respond to the following premises: they have to be elaborated with analytical criteria, be quantifiable and accepted by the scientific community, as well as using fundamental aspects such as naturalness, resilience, threat, floristic-phytocoenotic value and rarity. Other complementary criteria would represent the services that terrestrial plant communities provide to ecosystems and human societies, such as protection of the soil or water resources, as well as a coefficient that considers those ecosystems located near densely populated sites and that reflects a priority in their conservation. The sum of each of these fundamental values gives the biological value of each site. Additionally, each of the complementary criteria used as factors can be used to obtain the conservation interest of the site.

Bio-evaluation proposals and the application of indices of naturalness, risks and patrimonial value have existed since the 1980s. Lalanne, Bioret and Boullet (2016) [8], compiled them in their publication, which was additionally a tribute to Professor Jean-Marie Géhu, pioneer in the evaluation of the vegetation across the landscape as a characterization of the territory [19].

In the present study, we extended these management tools and assessed the management of protected areas by applying a new index, which we named DRA (acronym for Distinctiveness, Rarefaction and Area prioritization), and questioned whether the range of protection granted is in accordance with the conservation interest of the plant communities that these areas hold. Thus, we used three indices to obtain the DRA value: (1) habitat distinction, an estimator of syntaxonomic richness that can be used to measure impacts on the phytocoenotic diversity of a territory [20], in a way that greater richness and diversity of plant communities implies higher value of distinction; (2) degree of habitat rarefaction, used to compare habitat richness between two protected areas of different sizes [21]. This reduced the samples to a standard size, i.e., interpolating to the same number of plant communities: that of the protected area with the lowest abundance [22,23], thus making it possible to compare the conservation status of different natural areas with different surface areas, using the diversity and uniqueness of their plant communities; and (3) the level of prioritization of the protective measures which highlights those elements that are of greater importance for granting a level of protection to a natural area, considering the anthropic impacts on them [24]. This would allow for the checking of the degree of importance of vegetation in the assignment of a category of legal protection. More specifically, we assessed whether the status granted to these natural spaces is supported by the DRA values obtained for the plant communities they include and the values of the three described indices.

Thus, while the habitat distinction strengthens the exclusivity of certain plant communities of conservation interest, the degree of rarefaction complements it by incorporating the valuation of those elements that, being of high interest, are also less frequent (scarce). Finally, the level of prioritisation in the conservation of the area allows the identification of those that stand out because they incorporate the greatest number of communities. All of these considerations were integrated into the new proposed index (DRA), which therefore provides all the necessary information to question whether there is coherence with level of protection assigned and their plant communities value.

The usefulness and functionality of the DRA index were put to the test in the network of protected natural areas along the Andalusian coast. 

## 2. Results

### 2.1. Syntaxonomic Distinctness (STD)

**Figure 1:**
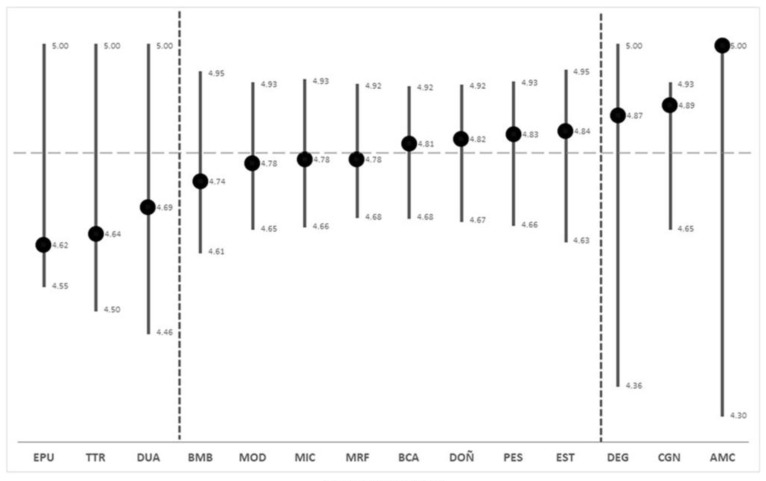
STD values (points) and the predicted interval between the maximum and minimum range (vertical lines) for each PA. AMC, Acantilados de Maro-Cerro Gordo; BCA, Bahía de Cadiz; BMB, La Breña y Marismas del Barbate; CGN, Cabo de Gata-Níjar; DEG, Desembocadura del Guadalhorce; DOÑ, Doñana; DUA, Dunas de Artola; EPU, Enebrales de punta Umbría; EST, Estrecho; MIC, Marismas de Isla Cristina; MOD, Marismas del Odiel; MRF, Marismas del Río Piedras y Flecha del Rompido; PES, Punta Entinas-Sabinar; TTR, Tómbolo de Trafalgar.

Based on the STD values obtained (Table S1), four quartiles were differentiated, distinguishing the first and the last, and integrating in a single group the intermediate ones, ordered from lowest to highest, including the expected probability ranges. This allowed for the distinguishing of three types of protected areas (PAs) (Figure 1): (a) Enebrales de Punta Umbría Natural Site (EPU), Tómbolo de Trafalgar (TTR), and Dunas de Artola (DUA) Natural Monuments. Lower values ranged between 4.6 and 4.7, and wide ranges between 4.4 and 5, the STD value below the median (dashed horizontal line); (b) Doñana National Park (DOÑ), Breña y Marismas del Barbate (BMB), Bahía de Cádiz (BCA) and Estrecho (EST) Natural Parks and Marismas del Odiel (MOD), Marismas de Isla Cristina (MIC), Marismas del Río Piedras y Flecha del Rompido (MRF) and Punta Entinas-Sabinar (PES) Natural Sites, with intermediate values between 4.74 and 4.84 and narrow ranges between 4.6 and 4.95; and (c) Cabo de Gata-Níjar Natural Park (CGN) and Desembocadura del Guadalhorce (DEG) and Acantilados de Maro-Cerro Gordo (AMC) Natural Sites, with very high distinction values between 4.87 and 5, very wide ranges between 4.3 and 5, and an STD value above the median. The communities found in CGN are grouped in a few syntaxons of higher hierarchical rank (order or alliance), hence the reduction in the probability rank.

### 2.2. Rarefaction Index (RRF)

**Figure 2:**
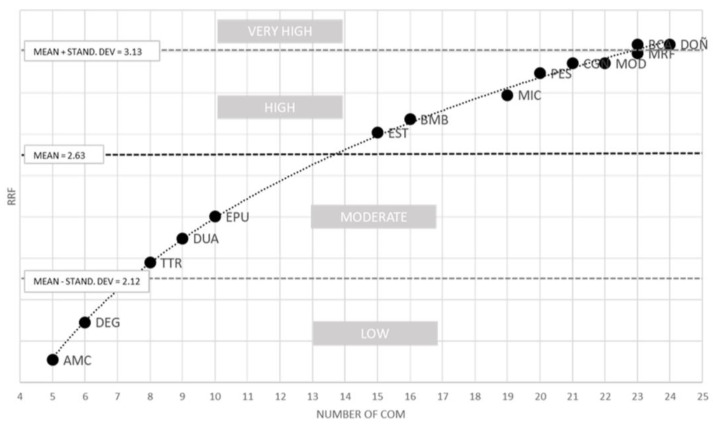
Ranking from the lowest to the highest rarefaction (RRF) value and number of communities in the Protected Areas. AMC, Acantilados de Maro-Cerro Gordo; BCA, Bahía de Cádiz; BMB, La Breña y Marismas de Barbate; CGN, Cabo de Gata-Níjar; DEG, Desembocadura de Guadalhorce; DOÑ, Doñana; DUA, Dunas de Artola; EPU, Enebrales de Punta Umbría; EST, Estrecho; MIC, Marismas de Isla Cristina; MOD, Marismas del Odiel; MRF, Marismas del Río Piedras y Flecha del Rompido; PES, Punta Entinas-Sabinar; TTR, Tómbolo de Trafalgar.

The results for the RRF index (Table S1) rendered four groups (ranges), considering the mean and the standard deviation (Figure 2). The first range contained AMC and DEG, which had a low RRF and few communities; the second range contained TTR, DUA and EPU, which had a moderate RRF and also consisted of few communities; and the third range contained EST, which had a high RRF but few communities, and BMB, MIC, PES, CGN, MOD and MRF, which also had a high RRF as well as many communities. Finally, the fourth range contained BCA and DOÑ, which had a very high RRF and many communities.

### 2.3. Priority Conservation Areas (PCA)

**Figure 3:**
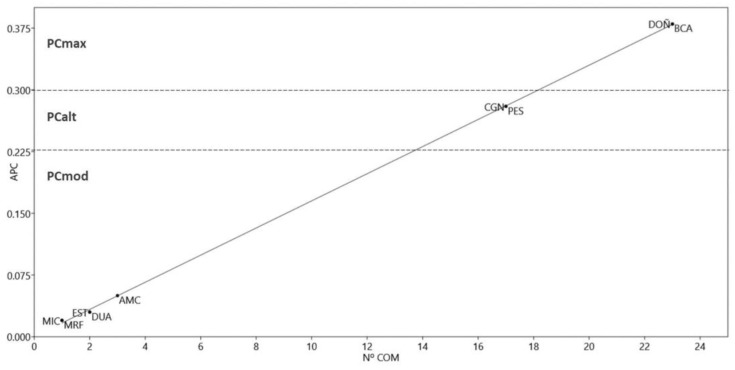
Distribution of Protected Areas (PAs) based on the values of the conservation priority index (PCA) and the number of communities (No. COM). PCmax, Highest priority; PCalt, High priority; PCmod, Moderate priority; AMC, Acantilados de Maro-Cerro Gordo; BCA, Bahía de Cadiz; CGN, Cabo de Gata-Nijar; DOÑ, Doñana; DUA, Dunas de Artola; EST, Estrecho; MIC, Marismas de Isla Cristina; MRF, Marismas de Río Piedra and Flecha del Rompido; PES, Punta Entina-Sabinar.

In the distribution (straight line) of PAs by Priority Conservation values (PCA; Table S1) and number of communities (Figure 3), four groups of PAs were distinguished: DOÑ and BCA, with the highest PCA values and many plant communities; CGN and PES, which had relatively high values; MIC, MRF, EST, DUA, and AMC, which had moderate PCA values and small surface areas. BMB, DEG, EPU, MOD, and TTR presented PCA values of 0 because their plant communities are represented in other PAs; i.e., the 16 BMB and eight TTR communities were also represented in BCA (23); the 10 EPU and 21 MOD communities are also present in DOÑ (23); DEG is a very small area, with only six communities, which are also present in many of the other PAs.

**Figure 4:**
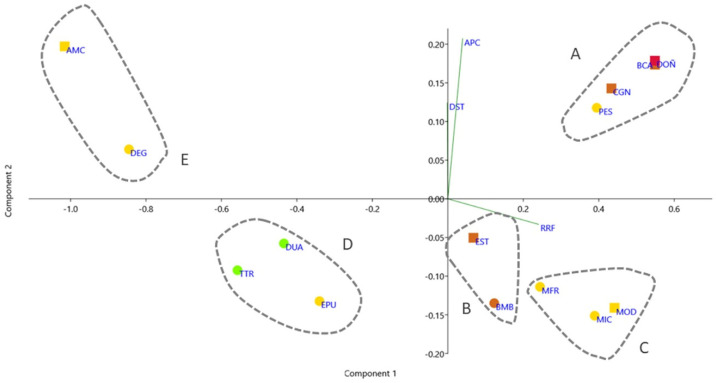
Principal Component Analysis of STD, RRF and PCA. National Parks are shown in red, Natural Parks in orange, Natural Areas in yellow, and Natural Monuments in green. Protected Areas considered Biosphere Reserves are shown in squares.

Principal component analysis of the STD, RRF and PCA showed that component 1 has a variance of 91.8% and that rarefaction is the variable with the highest weight (Figure 4 and Table 1). Five groups of PAs were clearly distinguished. Group A included DOÑ, BCA (closely linked by its communities to DOÑ) and CGN, in addition to PES (closely linked by its communities to CGN). Group B included EST and BMB, while Group C included MFR, MIC, and MOD, all of which had lower RRFs and STD, as they are closely linked to DOÑ. Group D included TTR and DUA, in addition to EPU. Finally, group E included AMC and DEG, both with a low number of communities (five and six, respectively), although in the case of AMC those present are almost exclusive to this area, hence its high STD and low RRF. In the case of DEG, the few communities present are found almost throughout the entire study area.

### 2.4. Correlation of the Legal Protection Index and DRA

The integration of the three indices into the new index DRA (acronym of STD, RRF and PCA) is shown in Table S1. We found a high correlation between the DRA and Legal Protection Index (LPI), which refers to the current protection status of a protected area (Section S1 and Table S1). However, it is worth noting the higher weight of the PCA and RRF with respect to it, with the STD being undervalued with the lowest correlation value (Table 2). 

**Figure 5:**
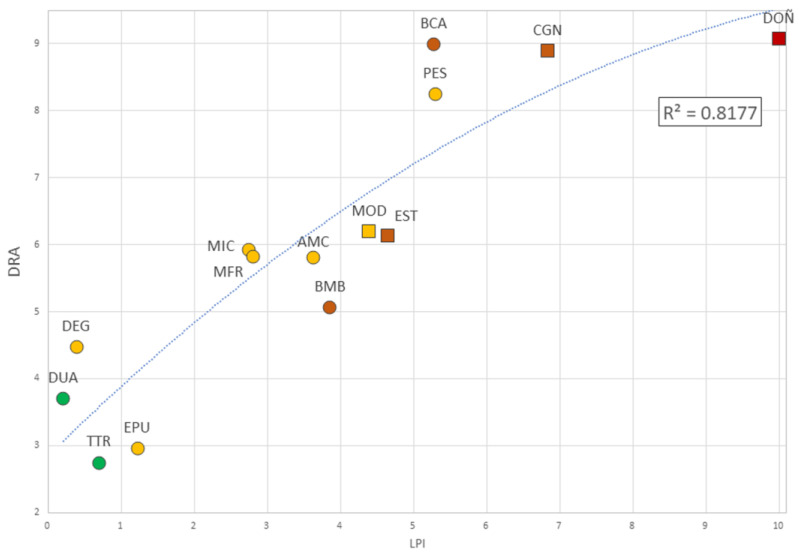
The Legal Protection Index [24] values compared to DRA. National Parks are shown in red, Natural Parks in orange, Natural Areas in yellow, and Natural Monuments in green. Protected Areas considered Biosphere Reserves are shown in squares.

The LPI and DRA values for each AP were distributed along a second-order polynomial trend line with R2 = 0.82 (Figure 5). The values for LPI, STD, PCA and DRA are shown in Table S1.

**Figure 6:**
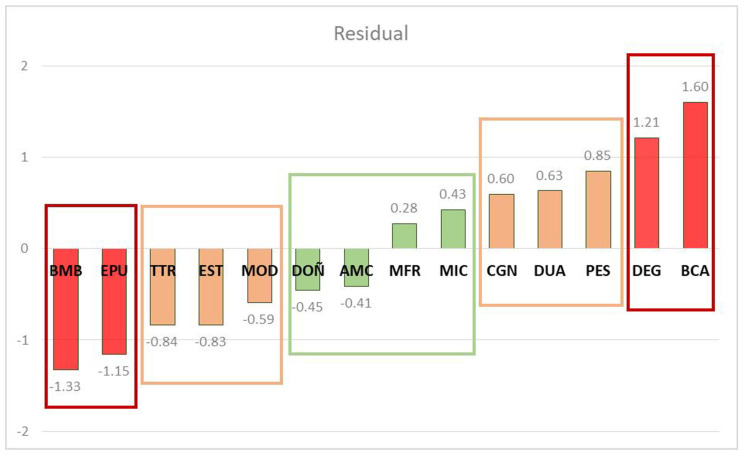
Plot of the residuals obtained for each PA after adjusting the curve obtained by comparing the values of the LPI with those of the DRA.

The residuals obtained for each PA after fitting the curve obtained by comparing the LPI with the DRA allowed three categories to be distinguished, based on their absolute values and their graphical interpretation (Figure 6):

(1) those residuals whose absolute value is >1 (red bars) corresponded to PAs with a significant divergence between LPI and DRA (DEG, BCA, BMB and EPU);
(2) a central range with absolute values between 0–0.50 (green bars), corresponding to those PAs where LPI and DRA showed a significant convexity (DOÑ, AMC, MRF and MIC);
(3) finally, those with absolute values between 0.51–1 (orange bars), which required a specific interpretation through the individual analysis of the ARD components (CGN, DUA, PES, MOD, EST and TTR).

**Figure 7:**
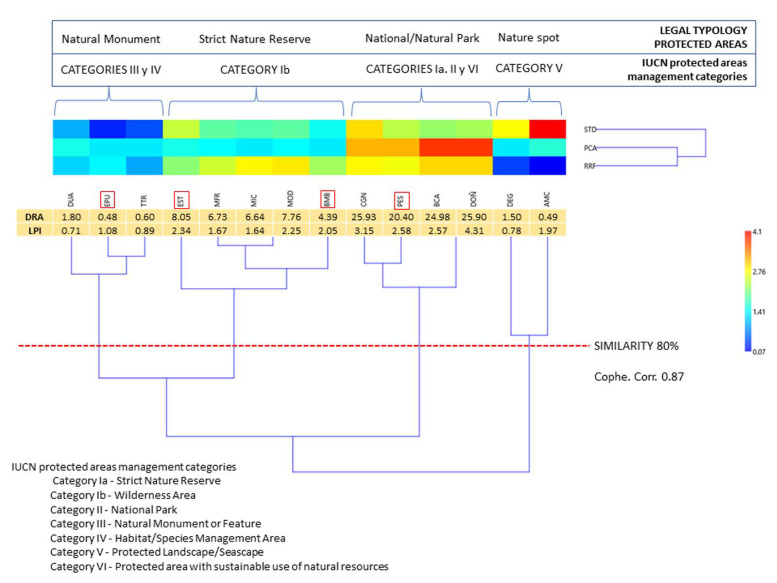
Comparison of the values of the Legal Protection Index (LPI) and the ARD Index proposed and the clusters derived from DRA values. Framed in red are those PAs whose assigned categories do not correspond to the DRA values obtained: EPU, EST, BMB and PES.

When clustering (Figure 7), the Phenon Line drawn at 80% similarity configured four groups, identified on the basis of DRA values, and consequently assignable to IUCN categories and/or those declared by the regional government of Andalusia (Spain) through the Andalusian Network of Natural Spaces [25].

## 3. Discussion

The World Commission on Protected Areas (IUCN-WCPA) [26], defined “protected area” as: A clearly defined geographical space, dedicated and managed, through legal or other effective means, to achieve the long-term conservation of nature with associated ecosystem services and cultural values, while specifying among other principles that the fundamental objective should be the conservation of nature, as well as maintaining or even increasing the degree of naturalness of the ecosystem being protected. It also establishes seven categories in a non-hierarchical system, to be applied in the context of national protected area systems and as part of the ecosystem approach: Ia—Strict nature reserve; Ib—Wilderness area; II—National park; III—Natural monument or feature; IV Habitat/species management area; V—Protected landscape; VI—Protected areas with sustainable use of natural resources. Each of these categories was described on the basis of a number of criteria (distinctive values, role in the landscape or uniqueness), although it acknowledges that the assignment to any given category depends more on the intended use by the management authority rather than on any fixed and unchangeable set of criteria. Such categorization is important because it works as the basis of the definition of the management objectives. This is even more the casewhen the allocation of categories has traditionally been the responsibility of governments, which has led to this situation being questioned. In any case, the IUCN’s own Guidelines for the Application of Protected Area Management Categories [26] recommend that “The only principle that should be applied in assigning categories is the appropriateness of the management objective assigned to the protected area within the system in relation to the ecological needs and threats to the species or ecosystem, in the context of the whole territory or marine environment in which the biodiversity occurs”.

More recently, the criteria for determining the areas to be protected and their categorization evolved and advanced with the help of conservation biology, technological tools such as geographic information systems and statistical processing based on biodiversity databases. The quality and beauty of the landscapes are no longer the only criteria for the selection of an area; the representativeness and complementarity that a reserve offers for the protection of biodiversity are now incorporated [27].

The method of inventorying the plant communities should integrate broad ecosystemic information, and it should allow for hierarchization using the vegetation association as a basic unit. Such a hierarchy would include the association in successive higher levels of alliances, orders and classes that would allow a dynamic definition of the territory. For this, we propose an integrated index (DRA). This cumulative index strengthens three variables for the proposal of PA management:

(1) Distinctness, understood as a measure of the diversity of a mosaic of ecosystems that distinguish a PA. It is increasingly recognized that appropriate measures of biodiversity within a particular taxonomic group should not merely be functions of the number of species and their relative abundance, but should also include information on the taxonomic relatedness of the species in question, which has led to the development of taxonomic distinctiveness (TD) [28]. On the same biological basis, syntaxonomy provides, through the hierarchization of communities and their treatment by the same method (STD), complementary information on the relationships of habitat diversity.

(2) Rarefaction, understood as the degree of rarity considering the extent of the PA. One of the challenges in determining the category of protection to be assigned to a territory is to resolve the size dependence and compare richness when the number of plant communities is not equal. To achieve this, one of the possible options [29] is the interpolation of species richness using the rarefaction technique, based on the shape of the species-abundance curve [30,31]. This traditional rarefaction was used to calculate the expected number of plant communities by reducing the samples to a standard size, i.e., interpolating into the same number of communities those areas with the lowest abundance [22].

(3) Prioritization, or understanding that the richness of communities confers a selective value in the process of choosing categories.

It should be noted that according to the results obtained in the clustering, in more than 70% of the cases there is an exact allocation of the categories with the values obtained from the DRA. Only four units differ in the value of the assigned category: PES is a Protected Site declared by the Regional Government, but its geographical proximity and degree of similarity in the cluster imply that it should be integrated as a Natural Park together with CGN, from which it is separated by the city of Almeria. Both BMB and EST are declared as Natural Parks; however, their coastal communities make them more similar to Natural Reserves. Finally, the low STD of EPU led to its integration as a Natural Monument, rather than a Natural Reserve as it appears in the RENPA.

The results of this study confirm that the use of the phytosociological method to describe plant communities in territories provides valuable information for the management of areas and their protection. Thus, statistical analysis using complementary diversity indices—such as STD, RRF and ACP—can be used to compare the status granted to PAs in territorial planning, using a new integrative index (DRA).

## 4. Materials and Methods

### 4.1. Materials

**Figure 8:**
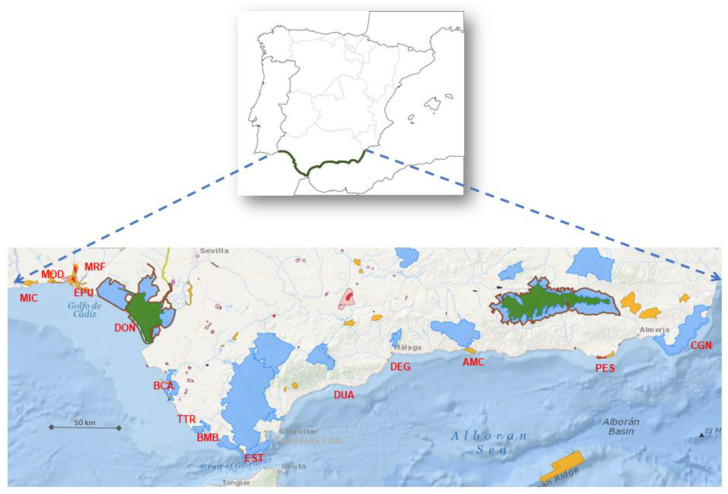
Location and biogeographical location of the Protected Areas studied on the Andalusian coast (Spain). AMC, Acantilados de Maro-Cerro Gordo; BCA, Bahía de Cádiz; BMB, La Breña y Marismas del Barbate; CGN, Cabo de Gata-Níjar; DEG, Desembocadura del Guadalhorce; DOÑ, Doñana; DUA, Dunas de Artola; EPU, Enebrales de punta Umbría; EST, Estrecho; MIC, Marismas de Isla Cristina; MOD, Marismas del Odiel; MRF, Marismas del Río Piedras y Flecha del Rompido; PES, Punta Entinas-Sabinar; TTR, Tómbolo de Trafalgar.

We selected the same 14 protected areas chosen by Salvo Tierra et al. (2020) [24] (Figure 8). These areas are included in the Andalusian Network of Natural Spaces. In line with the findings of Pereña (2018) [25], we created a syntaxonomic scheme of the set of plant communities observed that included their corresponding alliance, order, and class (Appendix A). Based on this information, we constructed a Basic Data Matrix (BDM) of the plant communities in each protected area (Table S2), and assigned an identifying abbreviation to each syntaxon.

Salvo Tierra et al. (2020) [24] calculated the Legal Protection Index (LPI) using a methodology based on the Protection Overlap Value. This methodology uses: (1) a protection status scale that is defined by the responsible administrative body and that overlaps in the same PA; and (2) the Legal Rigor, based on the number of overlapping protection regulations (Natural Resources Management Plans) within the same PA and their planning statutes (Master Plans for Use and Management).

Based on the results of the foregoing, numerical values were obtained for each Protected Area (Table 3). 

### 4.2. Method

We used three statistical diversity indices to determine whether certain areas have been granted their correct level of protection, based on their plant communities: syntaxonomic distinctiveness (STD), rarefaction (RRF) and level of prioritization of conservation areas (PCA). These were multiplied in order to obtain the DRA index. This was applied to the protected areas on the Andalusian coast and compared with their current category of protection.

So far, these three indices have always been applied to conventional taxonomy, but in this case, we further applied them to syntaxonomy because just as a taxon carries a pool of ecological, biogeographical, evolutionary, etc. information, syntaxons provide equally significant information. In this case, this is relevant because they are communities of plant species that converge in the same territory with identical ecological conditions.

### 4.3. Syntaxonomic Distinctness (STD)

Taxonomic structures are based on the number of taxa included at the highest levels of the taxonomic hierarchy therefore, in the case of using species as a unit, the higher hierarchical levels would be genus, family, order, subclass/class. They have been widely used in ecology to measure impacts on ecosystem diversity [20,28,32,33,34,35]. Among the estimators of taxonomic richness, taxonomic distinctness (TD [35] and references therein) is particularly significant because it is less influenced by sample size than by species diversity. Moreover, TD may be a more sensitive univariate index of community disturbance than species diversity [20]. Syntaxonomic structures provide relevant information, and thus have the same analytical potential as TD [36] for measuring impacts on territorial diversity.

Clarke and Warwick (1998) [28] proposed the ∆+ index (Delta-plus) to estimate TD. We extend this index to the syntaxonomic distinction (STD) of PAs. This index measures the overall average length of the syntaxonomic path between two randomly chosen PAs.

The ∆+ index for STD would be calculated according to the following equation:

(1) $$\Delta +=\left[\sum^{\;}\sum_{i<j}w_{ij}\right]/\left[s\left(s-1\right)\right]$$

where *s* is the number of phytosociological associations observed in a given PA, and *w_ij_* is the weight or distinction value given to each syntaxonomic branch of the hierarchical classification from association *i* to the first common node *j* (alliance), and so on up to the third and fourth level (order and class). 

### 4.4. Rarefaction (RRF)

The rarefaction (RRF) index was used to compare observed diversity between locations that have not been sampled equally [16]. To make a fair comparison of these sites, subsamples (i.e., communities) were drawn from the larger sample, and the expected richness in each unit (i.e., PAs) was calculated based on abundance distributions in the larger sample. The process was repeated for subsamples of different sizes. The final value of the RRF index showed the expected value of community richness according to the sample size of each PA [21]. This correction allows for the direct comparison of the richness of two samples that were originally of different sizes.

Hurlbert (1971) [31] proposed the following algorithm to determine the RRF:

(2) $$Sn=\sum_{i=1}^{S}\left(1-qi\right)$$

where *qi* = $\frac{\left(\frac{n-zi}{n}\right)}{\left(\frac{N}{n}\right)}$ would represent the probability that community *i* does not appear in a sample of size *n*, z_i_ is the number of *i* communities, and *(N/n)* is the binomial coefficient or the number of ways in which n out of N can be chosen.

This algorithm was calculated by applying an analytical solution known as “Mao’s tau”, in which standard deviations are converted to 95% confidence intervals [37].

### 4.5. Priority Conservation Areas (PCA)

The method proposed by Vane-Wright et al. (1993) [38] was used to determine the value of the PCAs. In a first cycle, the PAs with the most communities were selected. Subsequently, the communities within the PA were eliminated from the BDM. This procedure was repeated in successive cycles that included the remaining communities that had not been included in the PAs already selected. The values obtained in each cycle refer to the total number of communities in the study area.

With the values of these three variables (STD, RRF and ACP), a Principal Component Analysis (using the variance–covariance matrix method) was implemented to check the influence of each one of them on the different protected areas, based on their vegetation communities, as well as the one that presented a higher level of incidence in the way these areas are grouped.

The calculations of these three indices were carried out using PAST v. 4.11 software [39,40].

### 4.6. Calculation of the DRA Index

The proposed DRA index was established as a tool for assessing the suitability of the assignment of protection categories to natural areas. For this reason, it was calculated by means of the product of the values obtained and subsequently standardized from the DST, RRF and PCA indices, all of which were independent. In this way, the range of variation of the values obtained was adjusted to a similar range to that obtained for the LPI (Table S1).

DRA = DST_st_ ∗ RRF_st_ ∗ PCA_st_ (3)

The values obtained from the calculation of the three indices and DRA are correlated (Spearman’s non-parametric correlation) with the LPI, which is the reference index for the current protection status. 

DRA values were compared with those of the LPI (Table S1) to check how they are distributed along the trend lines of both. This was done with excel V. 2204. With the residues obtained, divergences between the DRA and the LPI in the different protected areas were checked using the “confidence tunnel” method [28].

Finally, in order to test the functionality of the new DRA index, a clustering of the PAs studied was carried out on the basis of the three components of the index and the current protection categories (LPI), taking into account the IUCN categories for the protection of areas and that of the Andalusian Network of Protected Spaces, and drawing a Fenon Line at 80% similarity. This was calculated with the PAST 4.1.1 software using the Euclidean distance and the WPGMA algorithm (co-phenetic correlation of 0.87).

## Figures and Tables

**Table 1:** Principal components analysis results.

PC	Eigenvalue	% Variance		PC1
1	0.287115	91.78	STD	−0.0061
2	0.018848	6.02	RRF	0.9869
3	0.006894	2.20	CPA	0.1610

**Table 2:** Spearman correlation values.

	LPI
DRA	0.82
STD	0.50
RRF	0.72
PCA	0.74

**Table 3:** Protected status of each Protected Area studied and Legal Protection Index value [24].

	DESIGNATED STATUS	AMC	BCA	BMB	CGN	DEG	DOÑ	DUA	EPU	EST	MFR	MIC	MOD	PES	TTR
Designated status at regional or national level	National Park						+								
	Natural Park		+	+	+		+			+					
	Natural Area	+				+			+		+	+	+	+	
	Natural Monument							+							+
	Natural Reserve													+	
Designated status by EU	Special Conservation Area	+	+	+	+		+		+	+	+	+	+	+	+
	Special Protection Area for Birds	+	+	+	+		+			+	+	+	+	+	
Designated status at international level	Biosphere Reserve				+		+			+			+		
	RAMSAR site		+		+		+						+	+	
	Specially Protected Areas of Mediterranean Importance	+			+										
	Geopark				+										
	World Heritage Site						+								
	Legal Protection Index	3.62	5.27	3.85	6.83	0.38	10	0.2	1.22	4.64	2.8	2.74	4.39	5.29	0.69

AMC: Acantilados de Maro-Cerro Gordo; Bahía de Cádiz; La Brea y Marismas del Barbate; Cabo de Gata-Níjar; Desembocadura del Guadalhorce; Doñana; Dunas de Artola; Enebrales de Punta Umbría; Estrecho; Marismas del Río Piedras y Flecha del Rompido; Marismas de Isla Cristina; Marismas del Odiel; Punta Entinas-Sabinar; Tómbolo de Trafalgar. Note: + indicates that the protected area has received this status.

## Data Availability

All data supporting the reported results are included in the paper.

## Supplementary Materials

The following supporting information can be downloaded at: https://www.mdpi.com/article/10.3390/plants12020406/s1, Table S1: Values of all calculated índices; Table S2: Distribution of communities by protected areas.

## Appendix A. Syntaxonomic Scheme of the Protected Areas [25]. Abbreviations of Plant Communities

*Cakiletea maritimae* Tüxen & Preising ex Br.-Bl. & Tüxen 1952 **Cakmar**
* Cakiletalia integrifoliae* Tüxen ex Oberdorfer 1949 corr. Rivas-Martínez, Costa & Loidi 1992 **Caklia**
* Cakilion maritimae* Pignatti 1953 **Cakion**
* Hypochoerido radicatae-Glaucietum flavi* Rivas Goday & Rivas-Martínez 1958 **HypGla**
* Salsolo kali-Cakiletum maritimae Costa & Mansanet 1981 SalCak*
*Cisto-Lavanduletea stoechadis* Br.-Bl. in Br.-Bl., Molinier & Wagner 1940 **CisLav**
* Stauracantho genistoidis-Halimietalia calycini* Rivas-Martínez, Lousã, T.E. Díaz, Fernández-González & J.C. Costa 1990 **StaHal**
* Coremation albi* Rothmaler 1943 **Coralb**
* Cisto salviifolii-Ulicetum australis* A.V. Pérez, Nieto & Cabezudo 1993 **CisSal**
* Fumano juniperinae-Cistetum crispi* Sánchez & Galán 1996 **FumCis**
* Halimio halimifolii-Stauracanthetum genistoidis* Rivas-Martínez 1979 **HalSta**
* Thymo albicantis-Stauracanthetum genistoidis* Galán, I. Sánchez & Vicente 1997 **ThyAlb**
*Crithmo maritimi-Limonietea* Br.-Bl. in Br.-Bl., Roussine & Nègre 1952 **Cretea**
* Crithmo maritimi-Limonietalia* Molinier 1934 **Cralia**
* Crithmo maritimi-Daucion halophili* Rivas-Martínez, Lousã, T. E. Díaz, Fernández-González & J. C. Costa 1990 **Cdnion**
* Limonietum emarginati* Asensi 1984 **Limemar**
* * *Crithmo maritimi-Limonion pseudominuti Molinier 1934* **Clnion**
* Crithmo maritimi-Limonietum malacitani* Diez Garretas 1977 corr. Diez Garretas 1981 **CriLim**
* Limonio cossoniani-Lycietum intricate* Esteve 1976 corr. Alcaraz, P. Sánchez, De la Torre, Ríos & J. Alvarez 1991 **LimLyc**
*Cytisetea-Scopario striati* Rivas-Martínez 1974 **CytSco**
* Cytisetalia scopario-striati* Rivas-Martínez 1974 **Cyalia**
* Retamion monospermae* Rivas-Martínez & Cantó 2002 **Retmon**
* Pycnocomono rutaefolii-Retametum monospermae* Pérez Chiscano 1983 **PycRet**
*Euphorbio paraliae-Ammophiletea australis* Géhu & Rivas-Martínez 2011 **EupAmm**
* Ammophiletalia australis* Br.-Bl. 1933 **Amalia**
* Ammophilion australis* Br.-Bl. 1921 **Amlion**
* Loto cretici-Ammophiletum australis* Rivas-Martínez 1965 corr. Rivas-Martínez, T.E. Díaz, Fernández-González, Izco, Loidi, Lousã & Penas 2002 **LotAmm**
* Honckenyo peploidis-Elytrigion boreoatlanticae* Tüxen in Br.-Bl. & Tüxen 1952 **HonEly**
* Cypero mucronati-Elytrigietum junceae* Br.-Bl. 1933 **CypEly**
* Euphorbio paraliae-Elytrigietum boreoatlanticae* Tüxen in Br.-Bl. & Tüxen 1952 **EupEly**
* Sporobolion arenarii* (Géhu & Géhu-Franck ex Géhu & Biondi 1994) Rivas-Martínez & Cantó 2002 **Spoare**
* Eryngio maritimi-Sporoboletum arenarii* (Arenes ex Géhu & Biondi 1994) Rivas-Martínez & Cantó 2002 **ErySpo**
* Sporoboletum arenarii* Rothmaler 1943 **Spoari**
* Crucianelletalia maritimae* Sissing 1974 **Crulia**
* Crucianellion maritimae* Rivas Goday & Rivas-Martínez 1959 **Cruion**
* Loto cretici-Crucianelletum maritimae* Alcaraz, T.E. Díaz, Rivas-Martínez & P. Sánchez 1989 **LotCru**
* Helichrysion picardii* (Rivas-Martínez, Costa & Izco in Rivas-Martínez, Lousã, T.E. Díaz, Fernández-González & J.C. Costa 1990) Rivas-Martínez, Fernández-González & Loidi 1999 **Hesion**
* Artemisio crithmifoliae-Armerietum pungentis* Rivas Goday & Rivas-Martínez 1959 **ArtArm**
*Halodulo wrightii-Thalassietea testudinum* Den Hartog ex Rivas-Martínez, Fernández Gonzalez & Loidi 1999 **HalTha**
* Thalassio-Syringodietalia filiformis* Borhidi, Muñiz & Del Risco in Borhidi 1996 **ThaSyr**
* Syringodio-Thalassion testudinum* Borhidi 1996 **SyrTha**
* Cymodoceetum nodosae* Feldmann 1937 **Cymnod**
*Juncetea maritimi* Br.-Bl. in Br.-Bl., Roussine & Nègre 1952 **Juetea**
* Juncetalia maritimi* Br.-Bl. in Br.-Bl., Roussine & Nègre 1952 **Jualia**
* Juncion maritimi* Br.-Bl. in Br.-Bl., Roussine & Nègre 1952 **Jucion**
* Elymo elongati-Juncetum maritimi* Alcaraz, Garre, Peinado & Martínez Parras 1986 **ElyJun**
* Polygono equisetiformis-Juncetum maritimi* J. C. Costa in J. C. Costa, Lousã & Espírito Santo 1997 **PolJun**

*Magnocarici elatae-Phragmitetea australis* Klika in Klika & V. Novák 1941 **MagPhr**
* Bolboschoenetalia compacti* Dahl & Hadac 1941 corr. Rivas-Martínez, Costa, Castroviejo & E. Valdés 1980 **Boalia**
* Bolboschoenion compacti* Dahl & Hadac 1941 corr. Rivas-Martínez, Costa, Castroviejo & E. Valdés 1980 **Bolion**
* Bolboschoeno maritimi-Schoenoplectetum litoralis* Br.-Bl. in Br.-Bl., Roussine & Nègre 1952 corr. Rivas-Martínez, Costa, Castroviejo & E. Valdés 1980 **BolJun**
*Nerio-Tamaricetea* Br.-Bl. & O. Bolòs 1958 **NerTam**
* Tamaricetalia* Br.-Bl. & O. Bolós 1958 **Tamlia**
* Tamaricion africanae* Br.-Bl. & O. Bolós 1958 **Tamafr**
* Polygono equisetiformis-Tamaricetum africanae* Rivas-Martínez & Costa in Rivas- Martínez, Costa, Castroviejo & E. Valdés 1980 **PolTam**
* Tamaricion boveano-canariensis* Izco, Fernández-González & A. Molina 1984 **Tambov**
* Inulo crithmoidis-Tamaricetum boveanae* Izco, Fernández-González & A. Molina 1984 **InuTam**
*Parietarietea judaicae* Rivas-Martínez in Rivas Goday 1964 **Paetea**
* Parietarietalia judaicae* (Rivas-Martínez 1960) Rivas Goday 1964 **Paalia**
* Lavaterion maritimae* Rivas-Martínez & Cantó 2002 **Lavmar**
* Rosmarinetum tomentosi* F. Casas & M. López in F. Casas 1972 **Rostom**
*Pegano harmalae-Salsoletea vermiculatae* Br.-Bl. & O. Bolós 1958. **PegSal**
* Salsolo vermiculatae-Peganetalia harmalae* Br.-Bl. & O. Bolòs 1954 **SalPeg**
* Salsolo oppositifoliae-Suaedion mollis* Rigual 1972 **SalSua**
* Frankenio laevis-Salsoletum vermiculatae* J. C. Costa in J. C. Costa, Lousã & Espírito Santo 1997 **FraSal**
* Withanio frutescentis-Lycietum intricate* Alcaraz, P. Sánchez, De la Torre, Ríos & J. Alvárez 1991 **WhiLyc**
*Posidonietea* Den Hartog 1976 **Poetea**
* Posidonietalia* Den Hartog 1976 **Poalia**
* Posidonion* Br.-Bl., Roussine & Nègre 1952 **Ponion**
* Posidonietum oceanicae* Funk 1927 **Posoce**
*Quercetea ilicis* Br.-Bl. ex A. & O. Bolòs 1950 **Quetea**
* Pistacio lentisci-Rhamnetalia alaterni* Rivas-Martínez 1975 **PisRha**
* Asparago albi-Rhamnion oleoidis* Rivas Goday ex Rivas-Martínez 1975
**AspRha**
* Cneoro tricocci-Buxetum balearicae* Rivas Goday & Rivas-Martínez 1969 **CneBux**
* Juniperion turbinatae* Rivas-Martínez 1975 corr. Rivas-Martínez 1987 **Juntur**
* Osyrio quadripartitae-Juniperetum turbinatae* Rivas-Martínez ex Rivas-Martínez, Lousã, T.E. Díaz, Fernández-González & J.C. Costa 1990 **OsyJun**
* Chamaeropo humilis-Juniperetum navicularis* Sánchez García, Sánchez Gullón, Linares Perea & Galán de Mera 2014 **ChaJun**
* Rhamno angustifoliae-Juniperetum turbinatae* Rivas-Martínez ex Freitag 1971 **RanJun**
* Rhamno oleoidis-Juniperetum macrocarpae* Rivas-Martínez 1965 **RolJun**
* Periplocion angustifoliae* Rivas-Martínez 1975 **Perang**
* Ziziphetum loti* Rivas Goday & Bellot 1944 **Zizlot**
* Rubio longifoliae-Coremation albi* Rivas-Martínez in Rivas-Martínez, Costa, Castroviejo & E. Valdés 1980 **Rution**
* Rubio longifoliae-Corematetum albi* Rivas-Martínez in Rivas-Martínez, Costa, Castroviejo & E. Valdés 1980 **RubCor**
* Querco rotundifoliae-Oleion sylvestris* Barbéro, Quézel & Rivas-Martínez in Rivas-Martínez, Costa & Izco 1986 **QueOle**
* Aro neglecti-Quercetum suberis* Rivas-Martínez & Díez-Garretas 2011 **AroQue**
*Rosmarinetea officinalis* Rivas-Martínez, T.E. Díaz, F. Prieto, Loidi & Penas 2002 **Rosoff**
* Anthyllidetalia terniflorae* Rivas Goday, Rigual, Esteve, Borja & Rivas-Martínez in Rivas Goday & Borja 1961 **Antter**
* Thymo moroderi-Sideritidion leucanthae* O. Bolòs 1957 corr. Alcaraz, T.E. Díaz, Rivas-Martínez & P. Sánchez 1989 **ThySid**
* Teucrio belionis-Helianthemetum scopulorum* Peinado, Martínez Parras, Alcaraz, Garre & de la Cruz 1985 **TeuHel**
*Saginetea maririmae* Westhoff, Van Leeuwen & Adriani 1962 **Sagmar**
* Frankenietalia pulverulentae* Rivas-Martínez ex Castroviejo & Porta 1976 **Fralia**
* Frankenion pulverulentae* Rivas-Martínez ex Castroviejo & Porta 1976 **Fraion**
* Parapholido incurvae-Frankenietum pulverulentae* Rivas-Martínez ex Castroviejo & Porta 1976. **ParFra**
* Hordeion marini* Ladero, F. Navarro, C. Valle, Marcos, Ruiz & M. T. Santos 1984 **Hormar**
* Hainardio cylindricae-Lophochloetum hispidae* Rivas-Martínez, Costa, Castroviejo & E. Valdés 1980 **HaiLop**
*Sarlicornietea fruticosae* Br.-Bl. & Tüxen ex A. & O. Bolós 1950 **Saetea**
* Limonietalia* Br.-Bl. & O. Bolós 1958 **Lialia**
* Limoniastrion monopetali* Pignatti 1953 **Limmon**
* Polygono equisetiformis-Limoniastretum monopetali* Rivas-Martínez & Costa in Rivas- Martínez, Costa, Castroviejo & E. Valdés 1980 **PolLim**
* Limonion confusi* (Br.-Bl. 1933) Rivas-Martínez & Costa 1984 **Limcon**
* Limonietum ferulacei* Rothmaler 1943 **Limfer**
* Lygeo sparti-Limonion furfuracei* Rigual 1972 **LygLim**
* Limonietum angustebracteato-delicatuli* Rivas-Martínez & Alcaraz in Alcaraz 1984 **Limang**
* Arthrocnemo macrostachyi-Suaedetalia braun-blanquetii* Rufo, Fuente & Sánchez Mata 2016 **ArtSua**
* Arthrocnemion glauci* (Rivas-Martínez in Rivas-Martínez & al. 1980) Rivas-Martínez & Costa 1984 **Artgla**
* Arthrocnemo macrostachyi-Sarcocornietum hispanicae* Fuente, Rufo, Teijeiro & Sánchez-Mata 2013 **ArtSar**
* Inulo crithmoidis-Arthrocnemetum macrostachyi* Fontes ex Géhu & Géhu Franck 1977 **InuArt**
* Salicornietalia fruticosae* Br.-Bl. 1933 **Sarlia**
* Sarlicornion fruticosae* Br.-Bl. 1933 **Sarion**
* Limonio cossoniani-Sarcocornietum lagascae* M.A. Alonso & De la Torre 2002 corr. Rufo et al. 2016 **LimSar**
* Sarcocornio pruinosae-Halimionetalia portulacoidis* Rufo, Fuente & Sánchez Mata 2016 **SarHal**
* Halimionion portulacoidis* Géhu 1976 **Halpor**
* Cistancho phelypaeae-Sarcocornietum pruinosae* Géhu ex Géhu & Géhu-Franck 1977 corr. Rufo et al. 2016 **CisSar**
* Sarcocornio perennis-Puccinellietum ibericae* J. C. Costa in J. C. Costa, Lousã & Espírito Santo 1997 corr. Rivas-Martínez, Fernández-González, Loidi, Lousã, Penas & Izco 2002 **SarPuc**
* Sarcocornion alpini* (Rivas-Martínez et al. 1990) Brullo et al. 2002 **Sarcon**
* Halimiono portulacoidis-Sarcocornietum alpini* Rivas-Martínez & Costa 1984 **HalSar**
* Sarcocornietum alpini* Br.-Bl. 1933 corr. Rivas-Martínez, Lousã, T. E. Díaz, Fernández González & J. C. Costa 1990 **Saralp**
* Suaedion verae* (Rivas-Martínez, Lousã, T. E. Díaz, Fernández González & J. C. Costa 1990) Rivas-Martínez, Fernández González & Loidi 1999 **Suaver**
* Cistancho phelypaeae-Suaedetum verae* Géhu & Géhu-Franck 1977 **CisSua**
* Frankenio corymbosae-Suaedetum verae* Alonso & De la Torre 2002 **FraSua**
*Spartinetea maritimae* Tüxen in Beeftink 1962 **Spetea**
* Spartinetalia maritimae* Conard 1935 **Spalia**
* Spartinion maritimae* Beeftink & Géhu 1973 **Spaion**
* Spartinetum densiflorae* Rivas-Martínez, Costa, Castroviejo & E. Valdés 1980 **Spaden**
* Spartinetum maritimae* Béguinot ex Corillion 1953 **Spamar**
*Thero-Salicornietea* Tüxen in Tüxen & Oberdorfer ex Géhu & Géhu-Frank 1984 **Thetea**
* Thero-Salicornietalia* Tüxen in Tüxen & Oberdorfer ex Géhu & Géhu-Frank 1984 **Tsalia**
* Salicornion patulae* Géhu & Géhu-Frank 1984 **Salpat**
* Suaedo spicatae-Salicornietum patulae* Brullo & Furnari ex Géhu & Géhu-Franck 1984 corr. Alcaraz, Ríos, De la Torre, Delgado & Inocencio 1998 **Suaspi**
* Suaedo splendentis-Salicornietum patulae* Rivas-Martínez, Costa, Castroviejo & E. Valdés 1980 corr. Rivas-Martínez 1991 **Suasal**
*Thero-Suaedetalia* Br.-Bl. & O. Bolòs 1958 **Sualia**
* Thero-Suaedion* Br.-Bl in Br.-Bl, Roussine & Nègre 1952 **Theion**
* Suaedo splendentis-Salsoletum sodae* Br.-Bl. 1933 **Salsod**
*Tuberarietea guttatae* (Br.-Bl. in Br.-Bl., Roussine & Nègre 1952) Rivas Goday & Rivas-Martínez 1963 em Rivas-Martínez 1978 **Tubgut**
* Cutandietalia maritimae* Rivas-Martínez, Díez Garretas & Asensi 2002 **Cutmar**
* Alkanno-Maresion nanae* Rivas Goday ex Rivas Goday & Rivas-Martínez 1963 corr. Diez-Garretas, Asensi & Rivas-Martínez 2001 **AlkMar**
* Wahlenbergio nutabundae-Loeflingietum pentandrae* Alcaraz, Díez Garretas & Asensi in Ferre, Díez-Garretas & Asensi 1985 **WalLoe**
* Linarion pedunculatae* Díez-Garretas, Asensi & Esteve in Díez-Garretas 1984 **Linped**
* Ononido variegatae-Linarietum pedunculatae* Diez-Garretas, Asensi & Esteve ex Izco, P. & J. Guitián 1988 **OnoLin**
* Triplachno nitentis-Silenetum ramosissimae* Peinado, Martínez Parras, Alcaraz, Garre & de La Cruz 1985 **TriSil**
* Malcolmietalia* Rivas Goday 1958 **Maalia**
* Anthyllido hamosae-Malcolmion lacerae* Rivas Goday 1958 em. Rivas-Martínez 1978 **AntMal**
* Linario donyanae-Loeflingietum baeticae* Rivas-Martínez, Costa, Castroviejo & E. Valdés 1980 **LinLoe**
*Zosteretea marinae* Pignatti 1954 **ZonMar**
* Zosteretalia* Béguinot 1941 **Zoalia**
* Zosterion* Christiansen 1934 **Zorion**
* Zosteretum noltii* Harmsen 1936 **Zosnol**
