# Supplementary material for: Application of Phytosociological Information in the Evaluation of the Management of Protected Areas
Source: Plants (Basel). 2023 Jan 15;12(2):406. doi: 10.3390/plants12020406 (PMC9863115; doi:10.3390/plants12020406)
Supplement: Supplementary file 1 [file plants-12-00406-s001.zip › plants-2097740-SI.pdf]

Table S1. Values of all calculated índices.

APs	LPI	STD	RRF	PCA	DRA
AMC	1.97	4.10	0.07	1.63	0.63
BCA	2.57	2.21	2.96	3.82	3.27
BMB	2.05	1.48	2.27	1.31	1.98
CGN	3.15	2.94	2.79	3.16	3.08
DEG	0.78	2.75	0.42	1.31	0.63
DOÑ	4.31	2.29	2.96	3.82	3.28
DUA	0.71	0.99	1.18	1.52	1.05
EPU	1.08	0.26	1.38	1.31	1.05
EST	2.34	2.45	2.15	1.52	2.08
MFR	1.67	1.91	2.50	1.41	2.26
MIC	1.64	1.83	2.79	1.31	2.46
MOD	2.25	1.91	2.87	1.41	2.57
PES	2.58	2.39	2.69	3.16	2.92
TTR	0.89	0.48	0.96	1.31	0.73

Abbreviations: AMC, Acantilados de Maro-Cerro Gordo; BCA, Bahía de Cádiz; BMB, La Breña y Marismas de Barbate; CGN, Cabo de Gata-Níjar; DEG, Desembocadura de Guadalhorce; DOÑ, Doñana; DUA, Dunas de Artola; EPU, Enebrales de Punta Umbría; EST, Estrecho; MIC, Marismas de Isla Cristina; MOD, Marismas del Odiel; MRF, Marismas del Río Piedras y Flecha del Rompido; PES, Punta Entinas-Sabinar; TTR, Tómbolo de Trafalgar. LPI: Legal Protection Index; STD: Syntaxonomic Distinctness; RRF: Rarefaction Index; PCA: Priority Conservation Areas; DRA: acronym for Distinctiveness, Rarefaction and Area Prioritisation.

Table S2. Distribution of communities by protected areas.

CLAS	ORD	ALLI	ASOC	AMC	BCA	BMB	CGN	DEG	DOÑ	DUA	EPU	EST	MIC	MOD	MRF	PES	TTR
Cakmar	Caklia	Cakion	HypGla				+									+	
Cakmar	Caklia	Cakion	SalCak		+		+	+	+	+	+	+	+	+	+	+	+
CisLav	StaHal	Coralb	CisSal		+												
CisLav	StaHal	Coralb	FumCis						+		+			+			
CisLav	StaHal	Coralb	HalSta		+	+											
CisLav	StaHal	Coralb	ThyAlb				+									+	
Cretea	Cralia	Cdnion	Limemar	+													
Cretea	Cralia	Clnion	CriLim				+										
Cretea	Cralia	Clnion	LimLyc									+					
CytSco	Cyalia	Retmon	PycRet			+				+							
EupAmm	Amalia	Amlion	LotAmm		+		+	+	+	+	+	+	+	+	+	+	+
EupAmm	Amalia	HonEly	CypEly				+	+		+						+	
EupAmm	Amalia	HonEly	EupEly		+	+			+		+	+	+	+	+		+
EupAmm	Amalia	Spoare	ErySpo				+									+	
EupAmm	Amalia	Spoare	Spoari							+							
EupAmm	Crulia	Cruion	LotCru				+			+						+	
EupAmm	Crulia	Hesion	ArtArm		+				+		+	+	+	+	+		+
HalTha	ThaSyr	SyrTha	Cymnod		+		+					+					
Juetea	Jualia	Jucion	ElyJun		+	+			+			+	+		+		
Juetea	Jualia	Jucion	PolJun				+		+					+		+	
MagPhr	Boalia	Bolion	BolJun				+									+	
NerTam	Tamlia	Tamafr	PolTam				+									+	
NerTam	Tamlia	Tambov	InuTam	+													
Paetea	Paalia	Lavmar	Rostom	+												+	
PegSal	SalPeg	SalSua	FraSal					+	+								
PegSal	SalPeg	SalSua	WhiLyc									+					

Poetea	Poalia	Ponion	Posoce	+			+										
Quetea	PisRha	AspRha	CneBux	+													
Quetea	PisRha	Juntur	ChaJun			+			+		+	+					
Quetea	PisRha	Juntur	OsyJun													+	
Quetea	PisRha	Juntur	RanJun		+	+			+		+	+		+	+		+
Quetea	PisRha	Juntur	RolJun		+												
Quetea	PisRha	Perang	Zizlot		+	+			+	+	+	+		+	+		+
Quetea	PisRha	Rution	RubCor		+	+			+	+	+	+	+	+	+		+
Quetea	Quelia	QueOle	AroQue				+										
Rosoff	Antter	ThySid	TeuHel		+	+					+	+	+	+	+		
Saetea	Lialia	Limcon	Limfer		+	+			+				+	+	+		
Saetea	Lialia	Limmon	PolLim				+									+	
Saetea	Lialia	LygLim	Limang						+				+	+	+		
Saetea	ArtSua	Artgla	ArtSar				+									+	
Saetea	SarHal	Sarcon	HalSar		+	+		+	+				+	+	+		
Saetea	ArtSua	Artgla	InuArt		+	+			+								
Saetea	SarHal	Sarcon	Saralp		+	+							+	+	+		
Saetea	SarHal	Halpor	CisSar		+	+			+		+			+	+		
Saetea	Sarlia	Sarion	LimSar				+										
Saetea	SarHal	Halpor	SarPuc		+	+			+		+			+	+		
Saetea	Sarlia	Suaver	CisSua		+	+			+					+	+		
Saetea	Sarlia	Suaver	FraSua		+	+			+				+	+	+		
Sagmar	Fralia	Fraion	ParFra						+								
Sagmar	Fralia	Hormar	HaiLop		+	+			+				+	+	+		
Spetea	Spalia	Spaion	Spaden		+	+							+	+	+		
Spetea	Spalia	Spaion	Spamar						+				+	+	+		
Thetea	Sualia	Theion	Salsod													+	
Thetea	Tsalia	Salpat	Suaspi		+				+				+	+	+		
Thetea	Tsalia	Salpat	Suasal										+		+		

Tubgut	Cutmar	AlkMar	WalLoe				+									+	
Tubgut	Cutmar	Linped	OnoLin		+			+		+		+			+		+
Tubgut	Cutmar	Linped	TriSil				+									+	
Tubgut	Maalia	AntMal	LinLoe						+								
ZonMar	Zoalia	Zorion	Zosnol				+										

Abbreviations: CLAS, class; ORD, order; ALLI, alliance. Note: + indicates the presence of those categories in that protected area.

Section S1. Description of Legal Protection Index (LPI).

In order to evaluate the "legal protection" of PAs, the "degree of legal protection" has been considered, on a decimal scale, based on the level of legal requirements and the protection effort in each figure [8]. Generally, the same protected area usually has more than one figure (overlapping) that reinforces the protective character, while at the same time it must have territorial management tools, such as the Natural Resources Management Plan (PORN) or the Use and Management Master Plan (PRUG).

The algorithm proposed by Salvo Tierra et al. (2020) [24] is used to calculate the Legal Protection Index (IPL):

$$LPI_{(0\text{-}10)} = (VSP * log10\ AP\ surface\ in\ Ha) * RL \qquad (4)$$

Where VSP (protection overlap value) would correspond to the sum of the values of the degree of legal protection, according to the previously established scale, of administratively declared figures that overlap in the same PA.

The RL (legal stringency) would be estimated using the following algorithm:

$$RL = nNP + (\frac{PORN}{2} + \frac{PRUG}{2}) \qquad (5)$$

Where nNP corresponds to the number of protective standards that overlap over a PA.
